# The social biography of antibiotic use in smallholder dairy farms in India

**DOI:** 10.1186/s13756-018-0354-9

**Published:** 2018-05-02

**Authors:** Abhimanyu Singh Chauhan, Mathew Sunil George, Pranab Chatterjee, Johanna Lindahl, Delia Grace, Manish Kakkar

**Affiliations:** 1 Public Health Foundation of India, Plot 47, Sector 44, Gurgaon, Haryana 122002 India; 2 Department of Public Health Sciences, Faculty of Medicine, University of Liège - Hospital District, Hippocrates Avenue 13 - Building 234000, Liège, Belgium; 3 Indian Institute of Public Health, Gurgaon, Haryana 122002 India; 4 Centre for Research and Action in Public Health (CeRAPH), University of Canberra, Building 22, Floor B, University Drive, Bruce ACT 2617 Australia; 5 Indian Council of Medical Research, Division of Epidemiology, National Institute of Cholera and Enteric Diseases, Kolkata, 700010 India; 6 International Livestock Research Institute, Nairobi, 30709-00100 Kenya; 7 Zoonosis Science Laboratory, Uppsala University, Po Box 582, Uppsala, SE-751 23 Sweden; 8 Department of Clinical Sciences, Swedish University of Agricultural Sciences, PO Box 7054, Uppsala, SE-750 07 Sweden

**Keywords:** Antimicrobial use, Antimicrobial resistance, Dairy farm, Dairy farmer, Veterinary, Qualitative, India

## Abstract

**Background:**

Antimicrobial resistance (AMR) has been identified as one of the major threats to global health, food security and development today. While there has been considerable attention about the use and misuse of antibiotics amongst human populations in both research and policy environments, there is no definitive estimate of the extent of misuse of antibiotics in the veterinary sector and its contribution to AMR in humans. In this study, we explored the drivers ofirrational usage of verterinary antibiotics in the dairy farming sector in peri-urban India.

**Methods and materials:**

The study was conducted in the peri-urban belts of Ludhiana, Guwahati and Bangalore. A total of 54 interviews (formal and non-formal) were carried out across these three sites. Theme guides were developed to explore different drivers of veterinary antimicrobial use. Data was audio recorded and transcribed. Analysis of the coded data set was carried out using AtlasTi. Version 7. Themes emerged inductively from the set of codes.

**Results:**

Findings were presented based on concept of ‘levels of analyses’. Emergent themes were categorised as individual, health systems, and policy level drivers. Low level of knowledge related to antibiotics among farmers, active informal service providers, direct marketing of drugs to the farmers and easily available antibiotics, dispensed without appropriate prescriptions contributed to easy access to antibiotics, and were identified to be the possible drivers contributing to the non-prescribed and self-administered use of antibiotics in the dairy farms.

**Conclusions:**

Smallholding dairy farmers operated within very small margins of profits. The paucity of formal veterinary services at the community level, coupled with easy availability of antibiotics and the need to ensure profits and minimise losses, promoted non-prescribed antibiotic consumption. It is essential that these local drivers of irrational antibiotic use are understood in order to develop interventions and policies that seek to reduce antibiotic misuse.

## Background

India is the global leader in the production of milk and dairy products, accounting for 18.5% of the global output, with an annual output of 146 million tons [1]. The tremendous growth in the demand for milk and other animal-source foods has been accompanied by a corresponding increase in small-scale ventures, characterised by farms that typically occupy less than one hectare, employ within-family labour, and function with minimal input costs by adopting intensive, industry style rearing of livestock [2]. Over 80% of all cattle holdings in India are in smallholder farms, which cover over 45% of agricultural land and account for over half of total production [2, 3].

India has witnessed unprecedented growth in the urban population over the past decade [4]. Peri-urban fringes, developing in the shadows of India’s growing cities, play an increasingly important role in ensuring food security including dairy farms [5]. To maintain production levels, these farms, which often function in jurisdictional grey zones, with minimal quality control, infrastructure, support and oversight, practise which may result in adverse public health impacts [6, 7]. One such practice, which may have long-term adverse effects, is the non-therapeutic, irrational use of antibiotics in farm animals [8, 9].

Antibiotics are arguably the single most important and widely used medical intervention of our times. They have been rampantly used not only in human medicine but also in agricultural system(s), specifically in livestock production. Antibiotics are used therapeutically to treat sick animals, as well as prophylactically and metaphylactically to prevent infection and as growth promoters [10]. Emergence of infectious agents which are resistant to commonly used antimicrobial agents threatens the advances made by modern medicine, and AMR organisms have rapidly become one of the primary public health challenges the world over, but especially in developing and low- and middle-income countries like India [11].

Multiple studies in India, beginning with exploratory studies in the 1980s, have consistently shown that a large proportion of the tested milk samples contain antibiotic residues [12–15]. A recent study undertaken in the organised as well as organised dairy farms has reported tetracycline, oxytetracycline, sulfadimidine and sulfamethoxazole above MRL in milk samples [16]. Similarly, antimicrobial residues was reported in 23.3% dairy farm in the settings similar to the current study [17]. However, there remains a dearth of evidence about the drivers and determinants of antibiotic use in dairy farms in India, especially with respect to vulnerable areas like peri-urban areas [18]. This study was conducted to understand the practices and drivers related to the veterinary use of antibiotics in peri-urban smallholder dairy farms in selected sites of India.

## Methods

### Study setting

The study was conducted among smallholding dairy farmers in peri-urban areas of Guwahati,east of India, (26.1445° N, 91.7362° E); Ludhiana, north of India (30.9010° N, 75.8573° E); and Bangalore, south of India (12.9716° N, 77.5946° E). Like any developing country, peri-urban areas of a typical city encompass a wide range of economic activities, including farming (dairy, poultry etc.), husbandry and, small and medium scale industries, land speculation, residential suburbanization and waste disposal [19]. Definition of Peri-urban is still context specific and varies from city to city, it’s difficult to estimate the exact population. However, large proportion of people from rural to urban migration settles in peri-urban fringes of the cities [19]. The background review of literature, the formative phase, and a formal consultation with experts enabled us to identify relevant stakeholders in each of the sites, whilst allowing us to refine the topic guides that we used for data collection. The main phase of data collection was preceded by a formative phase that included scoping interviews with key informants at each site, as well as a pilot testing of instruments. Fieldwork was carried out between1^st^ February 2015 to 30th September 2015 across all three study sites.

### Sampling and data collection

Data collection at each of the field sites was carried out in successive phases.. The dual strategies of purposive sampling and snowballing were employed to identify potential respondents with the help of the local partners in each of the field sites. This enabled us to not only identify those stakeholders whom knew to be relevant to this study, but also identify specific stakeholders at each site, who were involved, in some capacity, with smallholder dairy farmers (eg: traders and veterinary field assistants in Guwahati, informal treatment providers in Ludhiana, and the Karnataka Milk Federation officials in Bangalore). At each of the sites, we identified areas where most dairy farms were clustered and fitted the project definition of a smallholding dairy farm (A farm with up-to 10 cattle, at-least one milking and contributing to a minimum of 25% of the total family income). From this list we then selected farms that were spread across various locations, thus ensuring representation of farms from across the various clusters (i.e., north, south, east and west). Using a phasic, cyclical strategy for the fieldwork, data collection was stopped on reaching saturation point across the various key themes of inquiry.

The health related interviews were conducted by ASC (male) and MSG (male). Both the interviewers were practicing public health researchers with over five years experience in qualitative data collection and held graduation in public health (MPH) at the time of field work. Face to face interviews with farmers were conducted at their homes, whereas those with other stakeholders were typically conducted at their places of employment (like, veterinary hospital, pharmacy etc.). Local NGO facilitated the scheduling of interviews as per the time convenient to farmers. An appointment was sought with the government functionaries in advance and almost all interviews were conducted in the office premises. Most interviews with farmers and traders were conducted in the local language (Hindi, Punjabi and Kannada at Guwahati, Ludhiana and Bangalore site, respectively). However, government officials were comfortable in interacting in English. In order to ensure that the mediator did not introduce bias and followed the topic guide faithfully, mock interviews and training were carried out prior to the actual field visit. A typical interview lasted between 45 min to 1 h. All interviews were audio-recorded, transcribed, translated into English, and crosschecked against the original recordings.

### Data management and analysis

Data analyses was done using inductive approach and content analyses. The translated transcripts were then coded using the software package AtlasTi 7.2®, utilizing a reflexive and inductive approach to allow codes and categories to emerge from within the data. The initial list of codes was compared with newer codes, enabling refinement of the coding framework, which was utilised to guide the coding process. Coding was done by two coders and coding disagreements was sorted in consultation with senior researchers of the study (MK and DG).

In addition to the interview recordings, each researcher maintained detailed field notes in field diaries. This enabled capturing of details related to the key issues that emerged in each location, concerns regarding the fieldwork, as well as any potential trends that emerged from the participant responses. The field diary provided us with adequate details to discuss during the daily review carried out at the end of the day’s work and plan for subsequent data collection. The field diaries also helped in identifying early patterns as well as assessing attainment of saturation of responses.

At the end of each phase, data management and analysis of the previous site was completed, and summary results prepared. This enabled further probing of specific areas. This iterative process ensured that the data collected was grounded, rich in details, and saturation obtained prior to termination of data collection.

### Quality assurance

Interviews were conducted by trained investigators.They were monitored for completeness, correctness, and comprehensive transcription and translation of responses with appropriate labelling of recordings. Thirty per cent of the interviews from every site were randomly rechecked for transcription and translation. Due to inherent limitations of interpretation of qualitative data from different parts of the country, we undertook regular consultations with the steering group (comprising of seven experts across the fields of medical, veterinary and social sciences) about the data and its interpretations.

It was ensured that interviews were conducted in place where only interviewee and interviewer was present. The study followed the COnsolidated criteria for REporting Qualitative research (COREQ) for reporting the findings of this qualitative research study [20].

## Results

A total of 54 interviews (formal and non-formal) were conducted across the three sites (Table 1). These included dairy farmers, veterinary officers, veterinary field assistants, pharmacists, drug distributors and civic officials. Site-specific stakeholders were also identified through the snowballing process. Traders, who procured milk from farmers and sold to sweet shops and households, were interviewed in Guwahati. Officials of Karnataka Milk Federation (KMF), a cooperative with a membership base of around 90% of the smallholder dairy farmers in periurban Bengaluru, were also interviewed. Those who were approached, none of them refused to participate in the study.

**Table 1:** Details of the stakeholders interviewed

Study sites	Dairy farmer	Veterinary/ Ext. officer	Veterinary field assistant	Trader	Pharmacist/Drug Distr.	Civic official Or Union
Guwahati	7	5	3	3	3	3
Bangalore	4	6	2	N/A	2	3
Ludhiana	4	2	2	N/A	2	3

The results are presented as three core themes that emerged from insights of the different stakeholders: 1. Self-treatment and peer learning; 2. Limited systems support, outreach and oversight; 3. Limited regulatory framework to regulate use, market pressures and distribution of veterinary antibiotics. Details of sub-themes and domains are listed in Table 2.

**Table 2:** Core themes and sub-themes emerged from the inductive data analyses

Sl. No.	Domain	Core themes	Sub-themes
1	Community and Individual	Self-treatment and peer learning	Limited knowledge about antibiotics and their use
			Self-treatment using veterinary antibiotics
			Peer learning and self-treatment
2	Veterinary health system support	Limited system support, outreach and oversight.	Shortage of veterinary doctors
			Laboratory support to diagnose diseases and make informed prescription
			Support from extension services
			Shortage of pharmacists
3	Policy and market scenario	Limited regulatory framework on usage, market pressures and distribution of veterinary antibiotics	Absence of regulation for veterinary antibiotics
			Direct marketing of veterinary antibiotics to consumer
			Compulsion of milking - Market demand and competition.

Themes could be further grouped into drivers operating at three levels of the system: individual, health systems, and market or policy levels. The system, in this case, was defined as the smallholder dairy farm in periurban settings and its associated veterinary antibiotic use practices. The concept of a “system” allowed us to study the linkages and interactions between the sub-themes and core themes that operate at different levels. For the purpose of this study, individual/community level drivers were defined as practices at the level of farm owners, labourers, family members, community, traders and others players who can influence antimicrobial usage. Health systems drivers were defined as those associated with systems stakeholders like veterinary doctors, veterinary field assistants, laboratory staff and others who could possibly contribute to the dynamics of antimicrobial usage in small holding dairy farms. Policy level drivers included the drivers that were associated with the government, national as well as local, and policies affecting the use of antimicrobials in smallholding dairy farms.

### CORE THEME I - Self-treatment and peer learning: Individual and community level drivers for irrational usage of veterinary antibiotics

Self-treatment of animals by farmers and peer learning were significant determinants of antibiotic usage, which emerged as the core theme at the community/individual level. These core themes further comprised of limited knowledge, self-treatment, and peer-learning as sub themes.

#### Sub-theme I – Knowledge about veterinary antibiotics

Majority of the farmers across three sites were unaware of the word ‘antibiotic’. No local name existed specifically for the term either. Most of the farmers could not differentiate between antibiotics and non-antibiotic, allopathic medicines. However, the interviewed veterinarians reported that many farmers administer antibiotics to the farm cattle irrespective of the disease being infectious or not. Veterinarians also mentioned that choice of drug is based mostly on the ease of availability and the experience of the farmers with the drug while treating similar symptoms on previous occasions, some of which could be undertaken at the advise of a veterinarian. Fin.

#### Sub-theme II - Self-treatment using veterinary antibiotics

Most information on how farmers prefer medicines that give them ‘quick results’ came from pharmacists. Sick animals are treated with broad-spectrum antibiotics on the basis of prior experience. This experience could be that of the concerned farmer or be obtained through by social peer learning networks (like elders or influential farmers who had previously treated their cattle successfully; more details in the next subsection). Intergenerational transfer of this information also played a significant role. Additionally, nearly all the veterinarians reported that by the time a farmer brings his animal to a licensed veterinarian, the farmer would have already tried out several treatment strategies, none of which were successful in curing the affected animal.

*“We are trying this medicine for last three days but not seeing much improvement; we will wait a bit and see, and if we do not see any improvements, we will try to call a doctor.” (*Dairy farmer, Ludhiana).

*“If I know what the problem is then I try to manage it. Sometimes it will be the same problem that another cow had, so I will buy and give the same medicines that the doctor prescribed last time.” (*Dairy farmer, Guwahati).

One of the reasons stated for self-treatment was the cost of getting a veterinarian to come to the farm, especially in Guwahati. While this was not an issue among well-established farms, smallholding farmers found this to be barrier.

*“My biggest problem is if my animals fall sick. Getting a veterinarian to come to my farm is costly and I can’t afford it. I give the animal what I can”.* (Dairy farmer, Guwahati).

#### Sub- theme III - Peer learning and self-treatment

Many field veterinarians, as well as a few dairy farmers, reported that they follow the advice of the local elders, influential persons, or village heads (‘*Gaon budha*’ (Guwahati) and ‘*sarpanch’* (Ludhiana)) for advice related to medication. Most of these opinion leaders are commercial dairy farmers with farms having more than 50 heads of cattle. They usually enjoy a good relationship with pharmaceutical representatives and drug distributors.

### CORE THEME II - limited system support, outreach and oversight

Limited systems support, outreach and oversight were significant reasons of antibiotic usage and emerged as the core theme at the veterinary health systems level. These core themes further could be split into the following factors: a shortage of licensed veterinarians and a profusion of informal prescribers; laboratory support to diagnose diseases and plan appropriate therapeutic strategies; inadequate IEC (Information, Education and Communication) support through extension services; and a shortage of veterinary pharmacists with a profusion of informal drug distributors.

#### Sub-theme I: Shortage of veterinary doctors and active informal prescribers

All three levels of stakeholders in the three sitesreported that there was an acute shortage of trained veterinarians. Stakeholders, including veterinary doctors and state officials, also reported that veterinary field assistants and informal prescribers attempt to fill the gap. This results in a cadre of untrained caregivers with a propensity to overprescribe, leading to irrational prescription and usage of antibiotics. These informal prescribers are also known as ‘private doctors’ among the dairy farmers.

*“How it is possible for a doctor to visit 30,000 cows? So definitely, if you call me and someone else calls me at the same time and there is a distance of 10 KM between the two houses, it is not possible for me to attend to both the cases simultaneously. I will definitely have to send somebody to attend the other patient. So what I do is that I send the VFA and tell him to go and check on the patient, and consult me over the phone. Mobiles are extensively used now, so it is possible. I do not go for visits nowadays, yet I have the information that I need to know. I think non-availability* [of enough trained and licensed veterinarians] *is one of the reasons, and because of that, slowly they* (the VFAs) *are emerging as the first point of care consultant; the other reason is that if they call the VFA then the fees will be much lower than that of a veterinarian, I think that might also be one of the reasons”* (Veterinary doctor, Guwahati).

*“The private doctors, they keep visiting farms and are very busy. In fact, they are occupied from early in the morning to late evenings everyday. They are not really doctors but that’s what they are known as. They treat a lot of animals in these farms”* (Pharmacist, Ludhiana).

### Sub theme II: Laboratory support to diagnose diseases and make informed prescription

#### Senior government officials’ perspective

Across the three sites, senior officials in the animal husbandry department pointed out that labs and diagnostic support services were functional and provided value addition to the work of field veterinarians. However, when asked specifically about testing and screening facility for various infections in cattle, very few veterinarians mentioned regular testing done at the smallholder or commercial dairy farms. None of the state level officials reported any disease screening programs specifically directed at cattle in smallholder dairy farms.

#### Field veterinarians’ perspective

Veterinarians reported that they do not depend on lab reports to treat any sick animals. Most treatment plans were based on case history and symptomatic assessments. Lab support was only sought when the treatment administered to the animal did not give the desired results. Two reasons were stated by veterinarians for not seeking lab support: First, by the time a farmer reaches the veterinarian the farmer has already spent time and money in trying out other alternatives and the veterinarian has to begin some treatment almost immediately to save the cattle. Secondly, even if labs do exist, most of them are not adequately equipped; if they are equipped, it would be expensive and unaffordable to access their services, and hence it was not considered to be practical to utilize them.

*“If the lab is in working condition we don’t have a microbiologist, if the microbiologist is there then there is no proper equipment. So how do I make use of it? On paper it is all there but practically it is not possible. If I need a lab report, then I ask them to go to the university or to some private labs to get a report.”* (Veterinarian, Ludhiana).

*“Look, we treat primarily from the case history of the sick animal and after some years of experience you know that an animal which is in this condition, is suffering from this problem, and needs this treatment. Other than that not much to do for us.”* (Veterinarian, Guwahati).

#### Sub theme III: Support from extension services in context to veterinary use of antibiotics

##### Dairy farmers’ perspective

It was striking that none of the farmers across the three sites referred to benefitting from any extension services. They perceived services to be of poor quality. Also, according to them, these services were conducted more out of the need to demonstrate activities to students, and were not particularly concerned with the welfare of a farmer or their animals.

*“What services are you talking about? There is such a big college here and they can’t even provide us with proper semen.”* (Dairy farmer, Guwahati).

*“No we do not get anything from the department or college.”* (Dairy farmer, Ludhiana).

Many of those who attended demonstration sessions and meetings happened to be owners of commercial dairy farms. These were individuals who had no prior experience of dairying and had entered the sector recently.

*“The department does organise activities from time to time when they want to train their students. Other than that such activities are not focused on the small farmers and their farms.”*(Dairy farmer, Guwahati).

##### Extension department officials’ opinion

Extension departments are functional and claimed to offer relevant services to local dairy farmers on a regular basis. These services include sessions on the updated management practices in dairy farming, inputs on fodder management, disease management, breeding techniques, shed design, to name a few. Most services were offered completely free of charge so that local dairy farmers belonging to the lower socioeconomic strata could reap the benefits. However, when asked about the specific activities related to the use of antibiotics and their role in maintaining the health of animals, the interviewed stakeholders (state-level officials and veterinarians) failed to mention any particular programs.

*“Regular meetings are organized by the department and we have sessions taken by experts to give them* [dairy farmers] *the latest know-how on various issues on how to manage a dairy farm.”* (Senior extension department official, Ludhiana).

Many veterinarians reported that farmers often chose to not attend these sessions. According to them, this is due to the farmers’ belief and reliance on traditional knowledge, which is often handed down generations. In contrast, relatively new commercial dairy farmers, were more open to learning and behaviour modification in relation to dairy farming practices.

#### Sub-theme IV: Shortage of pharmacists and presence of informal drug distributors

Some veterinary officers and state level officials reported the scarcity of trained pharmacists in the peri-urban areas; this was especially notable in Guwahati. According to them, most of the drugs were distributed by drug distributors. Even if a pharmacist is present, prescription-based purchase is minimal in all the studied settings. They forwarded this as one of the reasons for the irrational use of veterinary antibiotics.

### CORE THEME III: Limited regulatory framework on usage and distribution of veterinary antibiotics, and market pressure: Policy and market level drivers

Limited legislative frameworks to regulate the use and distribution of veterinary antibiotics, and mitigate market pressures are significant determinants of antibiotic over usage. These emerged as the core theme at the policy level. These core themes could further be split into: absence of regulation for the prudent use of veterinary antibiotics, Direct-to-Consumer Marketing of Veterinary Antibiotics (DTCMVA) and compulsion to maintain productivity to meet market demands in the face of stiff economic competition.

#### Sub theme I: Absence of regulation for the prudent use of veterinary antibiotics

Nearly all veterinary doctors and senior state level officials expressed the need to deploy potent regulations to deal with the situation of antibiotic growth promoters and non-therapeutic use of antibiotics. Many of the senior officials also reported the absence of evidence-based guidelines related to the prudent use of veterinary antibiotics; even when they were present, they were constrained by the complete absence of a strategy to operationalize the recommendations and monitor their implementation.

#### Sub theme II: Direct to consumer Marketing of Veterinary Antibiotics (DTCMVA)

*“While visiting a village in Guwahati to capture the insights from small holder dairy farmers related to veterinary use of antibiotics, we observed a Medical Representative (MR) from a renowned pharmaceutical company visiting the most learned and influential (village leader) dairy farmer in the village. He was carrying boxes with a range of medicines - concentrates, calcium supplements as well as veterinary antibiotics. When asked about the content and why he has been keeping these items at the leader’s house, we were informed by the dairy farmers that this was the ‘drug depot’ of the village. All farmers could access and purchase these medicines as and when required. Pharmacies are too far away and a significant cost is incurred when visiting them. Older members of the village informed that the depot holder received financial incentives from the MR as well as a supply of medicines at a discounted rate. As the depot holder is comparatively more qualified and influential, dairy farmers often consult him for the medicines and treatment. We discussed and validated these responses with local veterinarian and state level officials.”* [Excerpt from field diary, 13th March 2015, Guwahati, Description of DTCMVA phenomena].

During field observations in Guwahati, we observed that the antibiotics are directly marketed in the village. The most influential or community leader or commercial dairy farmer acts as mediator between MR and smallholder dairy farmers. The relationship is mutually beneficial as the farmer gets the drugs at a discounted rate and without having to travel to a distant pharmacy; in return, this helps the pharmaceutical representatives to meet their sales targets. For the village elders or opinion leaders, this offers a position of relative power, which further consolidates their status in the community. Some smallholder farmers also reported that the depot holder was often offered medicines at a discounted rate by the pharmaceutical representative.

#### Sub theme III: Compulsion of milking - market demand and economic compulsion

Nearly all the stakeholders reported that the business of smallholding dairy farms operates at razor thin profit margins and to keep their livelihood intact the farmers need to maintain productivity in their animals on a daily basis. A majority of the farmers were unaware about the concept of withdrawal period following antimicrobial chemotherapy. However, even those who were aware of the importance of the withholding period reported that it was impractical due to high economic implications to their business. Additionally, famers also reported that they faced competition from milk cooperatives and private companies. This resulted in dwindling demands for non-packaged milk in the urban areas. As a result withholding milking and not supplying milk even for a day or two could result in an irreversible loss of customers which would have a significant adverse impact on their business. In absence of a formal system of incentives or disincentives, it was virtually impossible to practise the withholding. Health system level stakeholders also reinforced these concerns. According to them, withholding should be incentivised to prevent milk tainted with antimicrobial residues from entering into the food chain.

*“Who will pay for the milk I throw out?. Each cow gives an average of 10 litres of milk everyday. I have four milking cows, and if one is on treatment and milking is withheld, it translates into a loss of INR 300 minimum. We need to continuously feed the animal, irrespective of whether we are selling the milk or not. From where will I get money for this?” (Dairy farmer, Guwahati)*

*“We need to take appropriate measures. How can we expect a poor farmer to discard the milk? The Government should identify certain incentives so that these farmers comply with the policy.” (Senior official, Guwahati)*

*“Monetary loss for a day is just one way of looking at it. What about the customers we lose? Who is going to explain to them the reason behind why we are not providing the milk to them?” (Dairy farmer, Bangalore)*

### Behavioural model of drivers and determinants of non-prescribed use of antibiotics in smallholder periurban dairy farms

**Fig. 1:**
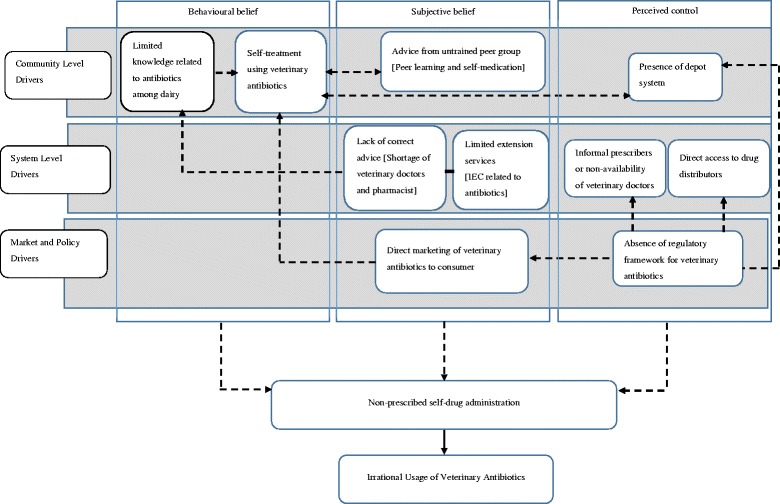
Behavioural model of drivers and determinants of non-prescribed use of antibiotics in small holder periurban dairy farms. Based on theory of planned behaviour, a diagrammatic representation of different individual, health system and policy level drivers affecting antimicrobial usage in small holder dairy farms

We developed a conceptual framework explaining the interplay of factors leading to non-prescribed usage of veterinary antibiotics using The theory of planned behaviour (Fig. 1) [21]. We mapped these factors to address the reasoned action for non-prescribed or self-administered use of veterinary antibiotics in the peri-urban smallholder dairy farms. This was determined by: [1] attitude towards irrational use of antibiotics, [2] subjective belief about the irrational use of antibiotics at the community level, and [3] the perceived control exerted over the act of irrational antibiotic use in livestock. Subjective belief is the perceived social pressure to perform or not to perform the behaviour and perceived control is an individual’s beliefs about the presence of factors that may facilitate or hinder performance of the behaviour. We classified the drivers operational at the community, the system, and the market and policy levels. These drivers, in combination, resulted in the practice of self-administered use of antibiotics in livestock. The conceptual model, shown in Fig. 1, indicates a closely knit, inter-related, web of factors with recursive and reversible relationships. It is essential to disrupt this chain at the critical linkages to make a meaningful reduction in the irrational and non-prescribed consumption of veterinary antibiotics in the peri-urban smallholder dairy farms of India.

## Discussion

This qualitative study adds to the growing body of evidence related to the issue of antimicrobial consumption. The study explicitly dealt with the non-prescribed and self-administered veterinary antibiotic usage in the smallholder dairy farms in peri-urban areas. Through in-depth-interviews across various stakeholders, this paper attempts to elucidate the complexities of antimicrobial usage in the smallholder dairy farming sector in peri-urban India. Each core theme was further explained under sub themes.

### Antibiotic use practices in small holder dairy farms

**Fig. 2:**
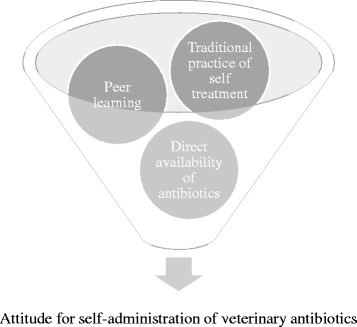
Attitude for self-administration of veterinary antibiotics. Diagrammatic presentation of Individual level drivers affecting antimicrobial usage in small holder dairy farms

In dairy farming, practices, knowledge and beliefs handed down from one generation to the next [22, 23]. Experienced dairy farmers have traditionally managed animal health issues using ethnoveterinary practices rooted in the use of indigenous medicinal herbs, utilising the Indian Systems of Medicine [24, 25]. The current generation has retained the self-reliance to manage livestock diseases on their own, and supplemented the traditional knowledge with their understanding of modern medicine. This self-reliance, in combination with market pressures and economic compulsions, has resulted in the widespread practice of self-administration of antibiotics in livestock for therapeutic, prophylactic and metaphylactic purposes, thus augmenting the risk of emergence of AMR (Fig. 2). Along with easy over-the-counter access to antibiotics, often without any prescriptions, or with invalid prescriptions, or with prescriptions from unlicensed practitioners, there is a fertile socioeconomic backdrop encouraging irrational use of antibiotics in livestock held by peri-urban smallholder farmers. Irrational use of antibiotics is further fuelled by veterinarians who are more influenced by social expectations than by scientific reasoning, as has been the case with human antibiotics prescribing practices [26].

### Drivers of antibiotic usage in Peri-urban smallholder dairy farms

The following key drivers directly influence the irrational usage of veterinary antibiotics: (a) direct marketing of veterinary antibiotics to consumer; (b) enabling of informal prescribers and caregivers to fill the gap created by inadequate coverage of veterinary services; (c) failure to regulate informal antibiotic supply chains in the community through drug depots and unfettered access to drug distributors; and (d) low literacy levels and poor awareness of antibiotics and the role they play in animal and human health.

#### a. Direct Marketing of Veterinary Antibiotics to consumer

**Fig. 3:**
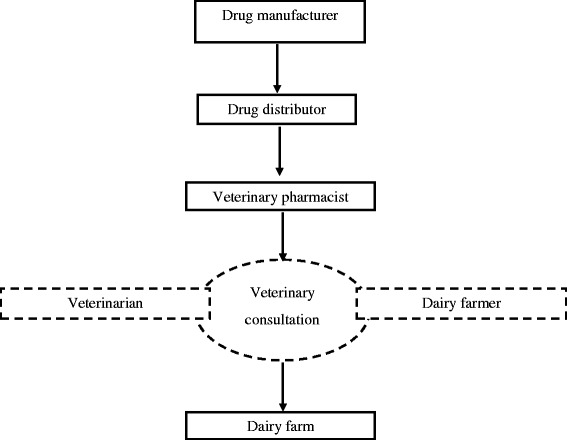
A typical formal drug distribution channel. Flow chart represents a formal and rational drug delivery system

**Fig. 4:**
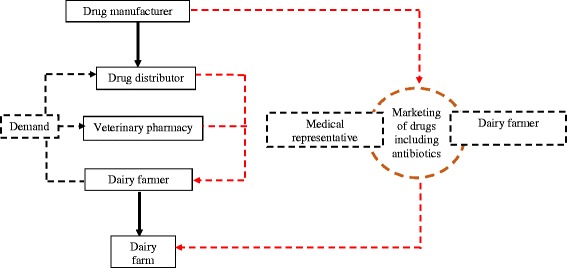
Informal channels of introduction of veterinary antibiotic in dairy farms

Although direct marketing and selling drugs without prescriptions is illegal in India, there are several loopholes in the existing regulatory provisions which have failed to keep up with the changing technological and socioeconomic milieu [27]. Direct-to-Consumer Pharmaceutical Advertising (DTCPA) is a heavily debated issue which remains strongly regulated and closely monitored in developed countries [28]. In the present study, some interesting facets with elements of DTCPA emerged as drivers of antibiotic use in livestock. Several veterinarians, pharmacists, and dairy farmers reported being approached by representatives of pharmaceutical companies advertising their products, some of which were veterinary antibiotics. Additionally, several respondents reported receiving free samples of the products. It is likely that bypassing the formal drug value chain (Fig. 3) by using informal channels of drug distribution, geared towards building a user base, further resulted in an irrational drug distribution (Fig. 4), increased the profit margins for the individual representatives who are usually required to meet time-bound sales targets [29, 30]. A previous study reported that the pharmaceutical companies do not impose the same influence in veterinary practice as in human [31]. However, the evidences from the current study strongly suggests pharmaceutical companies as potential influencer behind non-prescribed use.

#### b. Lack of qualified veterinarians and the role of informal caregivers

Use of veterinary antimicrobials without veterinarian consultation was reported in past with 87% and 38% among urban and rural farmers, respectively [32]. However, study did not report the drivers responsible for this. In the current study, a universal finding was the scarcity of trained veterinarians to cater for the animal health needs. There was significant convergence of all stakeholders on this matter. This is in concurrence with previous reviews on the veterinary capacity in the nation, which clearly demonstrated India’s constrained veterinary service delivery and the need to meet the scarcity through a systematic assessment of the human resources, both in terms of the number as well as the competence of the workforce, followed by the establishment of new veterinary colleges and other institutions to bridge the human resources gap [33–35].

In addition to the perceived shortage of veterinarians, in the present study, informal interactions also revealed that the veterinarians are more interested in private veterinary practice, which is oriented towards treating pet animals (Mostly, dogs, cats, rabbits and birds.), in contrast to the government sector facilities which had a stronger focus on livestock. This is likely to be a factor promoting the informal or unauthorized (para-vet/veterinary field assistants etc.) prescriber network, which endeavours to reach the underserved population. Similar finding was reported in an earlier study conducted among veterinary doctors in south eastern part of India [31]. Study reported that veterinary doctors perceives that prior prescription by unqualified prescribers was influencing antimicrobial prescriptions for animals. Recent study in northern India reported low level of knowledge among para-vet about antibiotic resistance and its public health impact [36]. The use of antibiotics not prescribed by a veterinarian, or the use of antibiotics from non-accredited sources was frequently reported in different parts of the world [37]. However, no single study has explained the interplay of different drivers at different levels potentially responsible for the non-prescribed usage of veterinary antimicrobials.

The shortage of trained and licensed pharmacists was also identified to be an issue by some state-level stakeholders. The pharmacist has a vital role to play in limiting access to antibiotics and providing proper guidance to care-seeking farmers. It is essential to incorporate them within the ambit of community-based antibiotic stewardship efforts to reduce the unintended consequences of overuse and abuse of veterinary antibiotics [38–40]. The absence of adequately trained pharmacists could contribute to the nexus between non-licensed distributors and representatives of pharmaceutical companies. According to a report based on a survey conducted in the WHO European Member States, adequately trained pharmacists can act as gatekeepers to rational drug usage interventions and are uniquely positioned to influence prudent antibiotic consumption [41]. The shortage of such a vital component of the healthcare delivery system, is, therefore, of special concern.

#### c. Easy access through informal antibiotic supply chains

The depot system, direct accessibility of community opinion leaders to pharmaceutical representatives, and easy access to over-the-counter antibiotics represents an informal supply chain through which the dairy farmers may access antibiotics and other veterinary drugs. The dairy farmers preferred this informal supply chain as well, since it provides direct access to medications perceived to be effective, without the accompanying costs, both in terms of money and time, of consulting a veterinarian. Similar findings were reported in a study from Peru where farmers preferred prescription as well as purchase of drug from other channels such as direct purchase from pharmacies and feed-store vendors [42]. Consequently, the farmers often contacted the pharmaceutical representatives directly when they needed to explore options for treating their animals.

Another phenomenon which raised concerns is the depot system. An elderly or experienced farmer, who often happened to be the opinion leader in the community, was approached by the pharmaceutical representatives, and provided sample medications to distribute to neighbouring farmers. The possibility remained that such informal routes, in addition to encouraging irrational use, could also promote multi-drug use or use of supra-therapeutic doses, as the elderly farmers would want to ensure clinical success and consolidate their position in the social hierarchy. A further consequence of the unregulated access to the supply chain of veterinary antibiotics is the repeated use of the same drug for different clinical conditions even if the underlying pathophysiology is different and warrants a different therapeutic approach.

#### d. Poor literacy, low level of education and low level of awareness

Access to information has been cited to be an important factor in promoting equity in healthcare [43, 44]. Building awareness about antibiotics has been identified as one of the key strategic objectives espoused by the World Health Organization (WHO) in its global action plan to contain AMR [45]. In the present study, an additional layer of complexity was added to irrational antibiotic use by peri-urban smallholder dairy farmers because of the relatively low levels of literacy and awareness, which has been previously reported by studies conducted in similar settings [46, 47]. This implies that the farmers might be unable to interpret complex medical information even if they have access to it. This assertion receives endorsement from the veterinarian input that farmers are often unable to comprehend why a drug is inappropriate for a given clinical scenario, even when symptoms mimic a previous episode where the same drug was prescribed.

The veterinarians also admitted that there is a definite “pull” from farmers who insist on antibiotics for illnesses where they may not be recommended; they preferred a shorter course of more expensive or newer antibiotics over a recommended, longer regimen. Similar findings were reported in a previous study from New Zealand, where 22% of the veterinary doctors admitted that their prescribing decision was influenced by non-clinical reasons such as farmers’ preferences [48]. Similarly, veterinarians reported ‘external pressures’, such as pressure from clients, legislation and public perception, strongly influence their antimicrobial prescribing behaviour [49]. Limited or no evidence is available to compare the findings in this context from India. Farmers interacted more often with pharmaceutical representatives or informal practitioners, who are likely to be more amenable to giving in to such demands. This could potentially lead to a vicarious pressure on veterinarians to prescribe per the farmers’ wishes to retain their patients. Longer duration of therapy could result in adverse economic implications for the farmers, hence, when such a course is recommended at government hospitals or licensed veterinarians, they risk losing their credibility with the farmers unless the farmers are adequately informed and counselled. These perverse financial forces could distort prescription practices even in the formal clinical systems, resulting in inappropriate use or overuse of antibiotics.

Though there is limited evidence related to the market pressures on smallholder dairy farmers contributing to compulsion to milking and overlooking the issue of withholding, the current study indicates that in the absence of quality-based incentives, farmers have no motivation to withhold milking cattle undergoing antimicrobial chemotherapy. Nearly all farmers reported a very small profit margin and the compulsion of milking to ensure solvency. There is a small, but growing body of published evidence, advocating for incentives to ensure milk quality. These incentives should be deployed in addition to IEC activities focussing on the improvement of mammary health, milk hygiene and safety was envisaged [50].

The lack of outreach activities, targeting the information needs of the community further deepened the crisis of misinformation in the farmers. This pattern of antibiotic misuse was further stimulated by the sense of control that most traditional dairy farmers felt they had on the farming process (unpublished findings, “Stakeholder mapping and analysis in peri-urban dairy farms of India”, Manish Kakkar). A typical finding was the creation of a depot system. This was seen to be a recursive issue, since the presence of a depot system encouraged farmers to self-administer antibiotics, and the depots were sustained by the continued interest of farmers in having quick access to medications, by-passing the somewhat onerous conventional animal healthcare system. This also created a perverse peer-support group, which functioned to propagate the misinformation about antibiotics and their utility, further ensuring their entrenchment in the community [51]. National Livestock Mission (NLM) could be a platform to engage the dairy farm owners to raise awareness related to prudent use of veterinary antimicrobials [52]. The mission has national presence and is supported by the central government. The current objective of the sub-mission in context to IEC involves increased awareness among all stakeholders involved in the animal husbandry sector regarding scientific methods of rearing, susceptibility to disease, vaccination, breed improvement, animal nutrition, schemes implemented by various agencies and support for Livestock Extension at various levels. An evidence-based intervention package related to knowledge on antibiotics, need for using prescribed antibiotics, adherence to prescribed therapy and observing withholding period can be incorporated in the revised NLM IEC strategies [52].

In addition to the centrally run schemes and missions, there are schemes under the state governments which aim to attract smallholders into the supply cycle to provide increased returns for their produce, thus stimulating production and encouraging the uptake of improved technologies. Inclusion of rational use of veterinary antibiotics as an objective under the awareness programme might result in changing the risk practices of dairy farmers with respect to veterinary antimicrobial consumption [53]. Similarly, other programmes like the National Programme for Bovine Breeding and Dairy Development (NPBBD), which was initiated in February 2014, could be used as a platform to raise awareness related to veterinary antimicrobial use among smallholder dairy farmers, as well as strengthening the laboratory screening facility of residues in [54].

## Limitations of the study

A very small number of state-level civic officials were involved in this study. Notwithstanding this, the limited number provided rich and meaningful data as the respondents who participated had decades of experience in animal husbandry and veterinary medicine. IDI with dairy farmers were performed in the local languages and then translated into English. Despite the rigorous verification process, some subtle nuances might have been missed during the verbatim transcribing.

## Conclusion

The current study identifies several factors which come together to determine the use of antibiotics in the smallholding dairy farms located in peri-urban fringes of Indian cities. The qualitative nature of the enquiry provides us with unique insights which are difficult to identify using the traditional quantitative surveillance approaches. In this study we explore the social biography of antibiotics, as they find their way through formal and informal routes, into the peri-urban smallholder dairy farms, often in the form of irrational, non-therapeutic, sub- or supra-therapeutic usage.

The study concludes that in the presence of weak veterinary care infrastructures with limited outreach activities, severe human resource limitations, poor legislative and regulatory oversight, and limited knowledge and awareness of the role of antibiotics in consumers, it would be difficult to combat the issue of emergent antibiotic resistance. Interventions such as community awareness programmes related to veterinary antibiotics, establishing an effective drug distribution policy, imposing penalties on defaulters, and strengthening of veterinary human resources both in terms of quantity as well as competence is required to address the issue adequately.

## Abbreviations

AMR: Antimicrobial Resistance
DTCMVA: Direct to Consumer Marketing of Veterinary Antibiotics
DTCPA: Direct to consumer pharmaceutical advertising
IEC: Information Education Communication
MR: Medical Representative
VFA: Veterinary Field Assistants
WHO: World Health Organization
